# *Out & Online*; effectiveness of a tailored online multi-symptom mental health and wellbeing program for same-sex attracted young adults: study protocol for a randomised controlled trial

**DOI:** 10.1186/1745-6215-15-504

**Published:** 2014-12-23

**Authors:** Jo-Anne M Abbott, Britt Klein, Suzanne McLaren, David W Austin, Mari Molloy, Denny Meyer, Bronte McLeod

**Affiliations:** National eTherapy Centre, Brain and Psychological Sciences Research Centre, Faculty of Health, Arts and Design, Swinburne University of Technology, Hawthorn, Victoria Australia; DVC-Research & Innovation Portfolio, School of Health Sciences and Psychology, and the Collaborative Research Network, Federation University Australia, Ballarat, Victoria Australia; National Institute for Mental Health Research, The Australian National University, Canberra, Australian Capital Territory Australia; School of Health Sciences and Psychology, Faculty of Health, Federation University Australia, Ballarat, Victoria Australia; School of Psychology, Faculty of Health, Deakin University, Burwood, Victoria Australia; Faculty of Health, Arts and Design, Swinburne University of Technology, Hawthorn, Victoria Australia; National eTherapy Centre, Faculty of Health, Arts and Design, Swinburne University of Technology, Mail H99, P.O. Box 218, Hawthorn, 3122 Australia

**Keywords:** eHealth, eMental health, Same-sex attraction, Young adults, Sexual orientation, Wellbeing, Mental health, Randomised controlled trial, Intervention, Cognitive behaviour therapy

## Abstract

**Background:**

Same-sex attracted young adults have been found to experience higher rates of mental health problems and greater difficulties in accessing specialist mental health care services compared to their heterosexual peers. Internet-based mental health interventions have the potential to be more engaging and accessible to young adults compared to those delivered face-to-face. However, they are rarely inclusive of lesbian women and gay men. Thus, the current study aims to evaluate the effectiveness of an online mental health and wellbeing program, *Out & Online* (http://www.outandonline.org.au), in comparison to a wait-list control group, for reducing anxiety and depressive symptoms in same-sex attracted young adults aged between 18 and 25 years.

**Methods/Design:**

We are recruiting, through media and community organisations, 200 same-sex attracted young adults with anxiety and/or depressive symptoms and mild to moderate psychological distress (Kessler-10 score between 16 to 21). Participants will be randomly allocated to the intervention (the online program) or the wait-list control group based on a permuted blocked randomisation method to allow for stratification by gender. Participants in the intervention group will receive a tailored program for up to three types of mental health difficulties simultaneously. The primary outcome of anxiety and/or depressive symptoms, and secondary outcomes related to psychological distress, wellbeing and health behaviour will be measured at pre-intervention (0 week), post-intervention (8 weeks) and at a 3-month follow-up (20 weeks).

**Discussion:**

This online mental health and wellbeing program will be one of the first online interventions to be designed specifically to be relevant for same-sex attracted individuals. If the program is found to be effective it will improve access to specialist same-sex attracted-relevant mental health services for young adults and will facilitate wellbeing outcomes for these individuals. This program will also be a significant development in the delivery of tailored interventions that target multiple types of mental health conditions simultaneously.

**Trial registration:**

Australian New Zealand Clinical Trials Registry: ACTRN12611000700932. Date registered: 7 July 2011.

## Background

Poor mental health has been identified as a key issue for same-sex attracted young adults (SSAYA). Research indicates that SSAYA are twice as likely to attempt suicide as peers with opposite-sex attractions [1–4], 3.5 times more likely to engage in self-harm and 5 times more likely to report suicidal ideation than their heterosexual peers [5]. SSAYA also experience more depressive symptoms compared with heterosexual youth [3, 6–10]. In addition, 9% of SSAYA have been found to meet criteria for the diagnosis of posttraumatic stress disorder (PTSD) [11], and social anxiety has been identified as a potential mental health problem among SSAYA as they learn to fear and avoid social situations [12].

The prevalence of mental health problems among SSAYA has been strongly linked to the experience of homophobia [13–15]. Homophobic attitudes give rise to prejudice, victimisation, physical and verbal abuse, harassment, and rejection of SSAYA within their home [16], school environment [17–19] and the wider community [11, 15]. These discriminatory behaviours result in stigmatisation, alienation and isolation among SSAYA, which, in turn, is related to depression, PTSD, social anxiety disorder (SAD) and suicidal ideation [20, 21]. In addition, the mental health of SSAYA can be influenced by internalised homophobia [14, 22], which refers to the extent that a same-sex attracted individual has internalised society's negative perceptions of homosexuality [23]. It has been proposed that internalised homophobia is experienced to varying degrees by almost all SSAYA raised in a society where heterosexuality is the sexual norm [16]. Internalising homophobic attitudes can lead to diminished self-concept and self-esteem [24]. Further, models of identity development propose that the adoption of a positive identity depends upon resolving internalised homophobia [25, 26].

In addition to higher rates of mental health problems compared to heterosexual youth and young adults, SSAYA also face greater barriers to accessing help such as feeling that mental health care services marginalise them by assuming heterosexuality and do not adequately assist them to address challenges unique to being same-sex attracted such as stigma and 'coming out' [27]. These barriers are intensified for SSAYA living in rural or regional areas where they have less access to relevant information, resources, support and services than their city peers. Evidence indicates that rural SSAYA attempt suicide six times more often than the population average [28] and experience higher levels of homophobia than their city peers [29]. Indeed, research with same-sex attracted adults living in rural areas indicates that most do not disclose their sexual orientation, that non-disclosure exacerbates social isolation, and that those who do disclose are vulnerable to homophobic responses from workers in the health care system [30].

### The role of the Internet for the delivery of mental health care

Given the difficulties that ‘at-risk’ SSAYA perceive in accessing mental health care services, particularly in rural and regional areas, the Internet may be an ideal way to deliver mental health interventions to this population [31]. The Internet has the potential to overcome a number of the structural and attitudinal barriers that often avert efforts to seek help, especially within rural and regional areas, including stigma, a shortage of trained health professionals and geographic and social isolation [32–35]. Previous research with adults, including young adults and those in rural and regional areas, has indicated that delivering interventions via the Internet provides flexibility, anonymity, and enhanced accessibility [36, 37]. Moreover, a web-based intervention is likely to be more engaging for young people who are early adopters of new technology [38]. Most young people, including those from disadvantaged backgrounds, have access to an Internet-enabled computer, with approximately 95% of 15 to 25-year-olds using the Internet [39].

Numerous Internet-based mental health interventions targeting young people have been developed and evaluated; for examples, see references [40–44]. These findings support the development and provision of Internet-based mental health programs for education about, and prevention and treatment of, a number of psychological symptoms and conditions experienced by young people [45]. In particular, online delivery of cognitive behaviour therapy (CBT) has been found to be effective in reducing symptoms of anxiety and depression [36].

Despite the evidence for online interventions and their potential for assisting SSAYA with mental health difficulties the majority are not specifically relevant to the needs of same-sex attracted individuals. In a review of 24 web- and mobile phone-based online interventions, Rozbroj *et al*. [27] concluded that online interventions seldom addressed the stressors faced by same-sex attracted individuals and contained content assuming or suggesting users were heterosexual.

### Online multi-symptom mental health and wellbeing program development

Given the need for online interventions relevant to SSAYA we developed an online CBT intervention (the *Out & Online* program, http://www.outandonline.org.au) designed to reduce anxiety and depressive symptoms and enhance wellbeing in SSAYA aged 18 to 25 years. The program was made relevant to SSAYA by using inclusive language and content (for example, pictures and videos depicting people in same-sex relationships), applying some of the strategies to stressors unique to being same-sex attracted (for example, 'coming out', feeing accepted by heterosexual peers and enhancing self-esteem and self-acceptance in the face of homophobia and internalised homophobia), and providing information on support services specialising in supporting same-sex attracted individuals.

### Study aims

The present study aims to evaluate via a randomised controlled trial (RCT) the *Out & Online* program in SSAYA aged between 18 and 25 years recruited via media and community organisations. The program will deliver symptom-relevant material across up to three different types of mental health difficulties (out of generalised anxiety, social anxiety, posttraumatic stress, obsessions and/or compulsions, panic and depressive symptoms) so as to present individual users with a customised mental health early intervention that respects their individuality, and acknowledges the reality and frequency of comorbid symptomatology.

The primary aim of the study was to examine the effectiveness of the *Out & Online* program for reducing anxiety and depressive symptoms and improving wellbeing. It was anticipated that additional benefits participants might gain from the program would include reduced internalised homophobia, more positive attitudes towards seeking help, greater sense of control over their health and greater satisfaction with their lives.

## Methods/Design

### Study design

**Figure 1:**
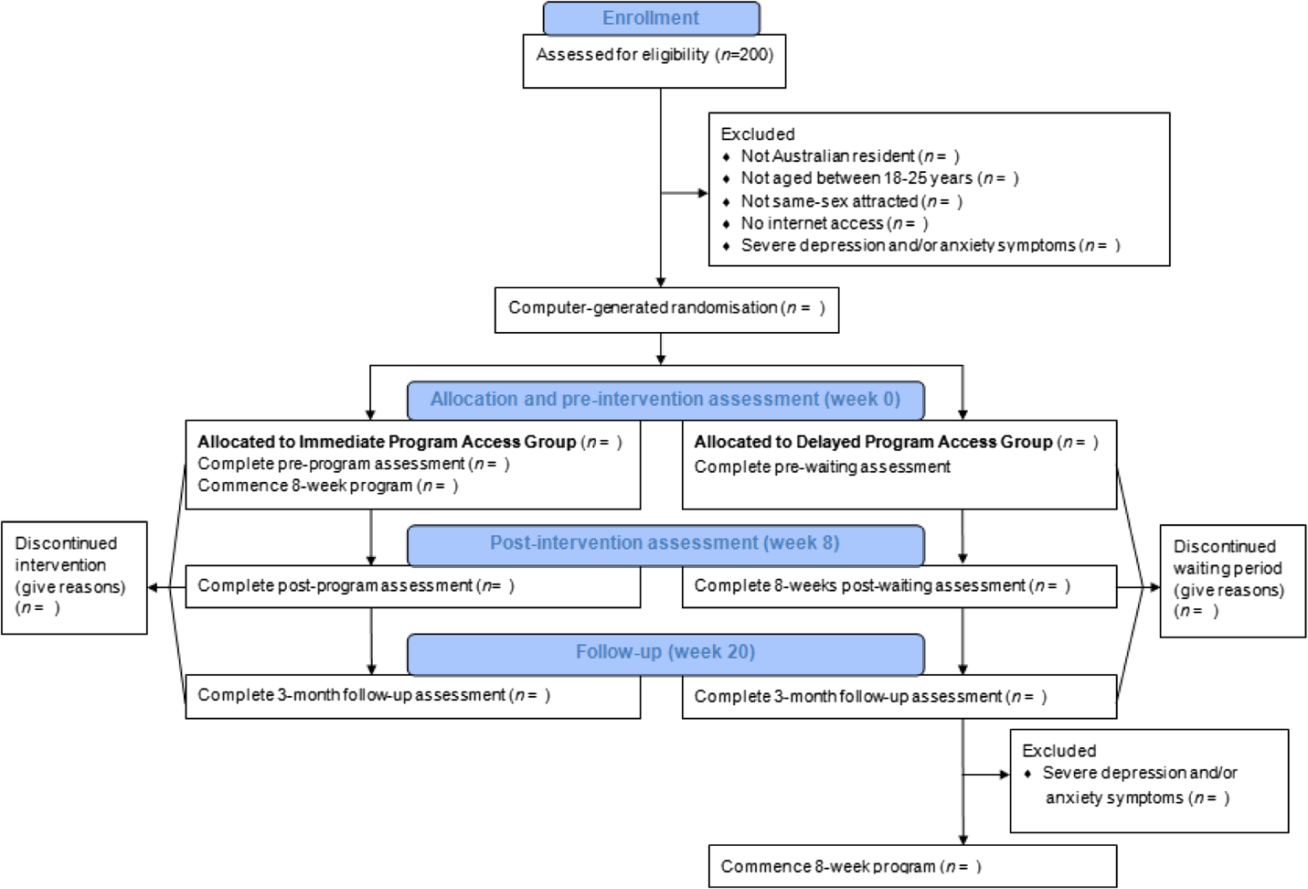
**Flow chart of the study design.**

This study will evaluate the intervention using an RCT. Participants meeting inclusion criteria and who have provided informed consent to participate will be randomly allocated to receive the intervention immediately or after a 20-week waiting period. Primary and secondary outcomes will be assessed via online surveys obtained from participants at pre-intervention (week 0), while completing the online program (weeks 2, 4 and 6), post-intervention (week 8) and 3-month follow-up (week 20). Figure 1 shows a flow chart of participant involvement in the RCT.

### Participants

We will recruit participants through Australian organisations that work with SSAYA, mental health organisations, advertising via social, online and news media, and information sessions to community groups (including support groups for SSAYA, health professionals that work with SSAYA).

Inclusion criteria are: Australian resident, aged between 18 and 25 years, same-sex attracted (self-identify as gay, lesbian or bisexual), access to the Internet, mild to moderate psychological distress (Kessler-10 score between 16 and 21) and having depressive and/or anxiety symptoms (being eligible to receive tailored content for at least one anxiety or depressive symptom).

### Intervention

To inform the development of the program, focus groups were conducted with SSAYA (aged 18 to 25 years) who discussed their experiences regarding their sexual orientation, their mental health needs and their preferences for support services. These data, along with expert review, informed the modification of existing online CBT-based prevention and treatment programs of the National eTherapy Centre. The program content was adapted to be suitable for integrated multiple symptom treatment. In addition, content was personalised for SSAYA, by using inclusive language and content (for example, use of pictures and videos depicting people in same-sex relationships), providing examples of stressors, unhelpful thoughts and beliefs and unhelpful behaviours unique to challenges associated with being same-sex attracted (for example, 'coming out', feeling accepted by heterosexual peers, fear of being assaulted for being gay), providing information on services specialising in supporting same same-sex attracted individuals, providing information on how self-esteem can be affected by being same-sex attracted (for example, through homophobia and internalised homophobia) and strategies to strengthen self-esteem and self-acceptance. Further refinement of the program was undertaken following usability testing, whereby SSAYA reviewed specific modules within the program and made recommendations for amendments to content. This feedback was incorporated where appropriate.

The *Out & Online* program was developed as a standalone resource that should complement face-to-face therapy, but in itself does not require the involvement of a health professional. The program contains seven brief modules containing mental health and wellbeing information and exercises based on CBT principles. The modules contain generic information and information and exercises specific for the symptoms participants endorse in an online assessment at pre-intervention, which screens for generalised anxiety, obsessions and compulsions, posttraumatic stress, specific fear, social anxiety and depressive symptoms. Some program content is also tailored for gender. It is recommended to participants that they spend an hour a week over eight weeks reading through the online modules and practising offline exercises. An eighth module on prevention and help for suicidal thoughts is accessible to all participants at all times.

### Wait-list control group

The intervention is being compared to a wait-list control group so that participants in this group have the opportunity to complete the program. Participants in the wait-list control group will receive access to the intervention program after a 20-week delay. They are able to access any other mental health and wellbeing services during this time.

### Randomisation

An automated computerised randomisation system will generate random sequences for group allocation. This will be based on a permuted blocked (block size = 10) randomisation method to allow for stratification by gender, ensuring that equal gender numbers are allocated to each of the two conditions. Trial enrolment, randomisation and assignment to intervention will all be automated and will not require any human input.

### Administration of assessments

Upon registering on the *Out & Online* website participants will complete baseline assessments (week 0). Automated Email prompts will be sent to participants asking them to complete online assessments at 8 and 20 weeks post-randomisation. In addition, while completing the online program, participants will be prompted by automated Email to complete a survey at weeks 2, 4 and 6 of the 8 week program. Table 1 shows the schedule of assessments for each time point.

**Table 1:** **Intended assessments and administration frequency**

Outcomes	Concept	Instrument	Items	Intervention group			Wait-list control group			
				Week 0	Week 8	Week 20	Week 0	Week 8	Week 20	Week 28
Primary	Depressive symptoms	Patient Health Questionnaire-9	9	x	x	x	x	x	x	x
	Generalised anxiety	Generalised Anxiety Disorder-7	7	x	x	x	x	x	x	x
	Panic	Panic Disorder Severity Scale	7	x	x	x	x	x	x	x
	Social anxiety	Social Phobia Inventory	17	x	x	x	x	x	x	x
	Obsessions and compulsions	Obsessive-Compulsive Inventory-Revised	18	x	x	x	x	x	x	x
	Posttraumatic stress	Posttraumatic Stress Disorder Checklist-Civilian Version	11	x	x	x	x	x	x	x
	Specific fear	Fear Questionnaire	16	x	x	x	x	x	x	x
Secondary	Psychological distress	Kessler-10	10	x	x	x	x	x	x	x
	Psychological functioning	*Out & Online* Symptom eScreener	7	x	x	x	x	x	x	x
	Internalised homophobia	Internalised Homophobia Scale-Revised	5	x	x	x	x	x	x	x
	Help-seeking attitudes	Attitudes Toward Seeking Professional Psychological Help Scale	10	x	x	x	x	x	x	x
	Life satisfaction	Satisfaction With Life Scale	5	x	x	x	x	x	x	x
	Locus of control	Multidimensional Health Locus of Control Scales-Form C	18	x			x			
	Wellbeing	Mental Health Continuum Short Form	14	x	x	x	x	x	x	x
Moderators	Demographic factors	*Out & Online* Demographic Questionnaire	24	x			x			
Treatment	Program satisfaction	Intervention Evaluation Questionnaire	14		x					
	Departure	Departure Questionnaire	9							

Note*:* in addition to the above schedule, the Kessler-10 will be administered at fortnightly intervals during program completion.

### Primary outcomes

The primary outcomes being assessed are reduction in anxiety and/or depressive symptoms. Validated questionnaires will be used to measure generalised anxiety (Generalised Anxiety Disorder 7 [46]), panic (Panic Disorder Severity Scale [47]), obsessions and compulsions (Obsessive-Compulsive Inventory-Revised [48]), social anxiety (Social Phobia Inventory [49]), posttraumatic stress (Posttraumatic Stress Disorder Checklist-Civilian Version [50]), specific fear (the Fear Questionnaire [51]) and depressive symptoms (Patient Health Questionnaire-9 [52]). These measures are also being used to determine whether participants are eligible to receive specific symptom content in their tailored online program.

In addition, an online screening device (eScreener) was developed in 2011 to measure anxiety and depressive symptoms. This eScreener will be compared against the validated questionnaires in order to develop a shorter means of determining symptom content for the online program. Once the eScreener is validated future entry to the program will first involve an assessment of psychological distress (with the Kessler-10 scale; K10 [53, 54]), with alternate referral recommendations with people with little or very high distress, and then administration of the eScreener as the sole means of determining symptom relevant content.

### Secondary outcomes

As each participant will receive a different number (between one and three) and combination of symptom content (out of generalised anxiety, panic, social anxiety, obsessions/compulsions, specific fear and depressive symptoms), the K10 [53, 54] will be used as a secondary outcome to evaluate progress at fortnightly intervals during the intervention, and at pre- and post-intervention. As research has revealed a strong association between K10 scores and a current Composite International Diagnostic Interview diagnosis of anxiety and affective disorders [54], this short scale is appropriate for the fortnightly progress evaluations and it will allow us to examine the influence of different program lengths and types on the main outcomes of interest.

Additional secondary outcomes will include life satisfaction (Satisfaction With Life Scale [55]), wellbeing (Mental Health Continuum Short Form [56]), internalised homophobia (Internalised Homophobia Scale-Revised [57]), help-seeking attitudes (Attitudes Toward Seeking Professional Psychological Help Scale [58]) and locus of control (Multidimensional Health Locus of Control Scales-Form C [59]). Demographic and background information will be collected at pre-intervention. Participants in the intervention group will also complete a program evaluation survey, which will include standard items regarding satisfaction with the online intervention.

Participants will have the option to 'opt-out' of the trial by clicking on a link on the website or automated Email and those that do this will be invited to complete a Departure Questionnaire which captures reason for opting out of the study.

### Hypotheses

It is hypothesised that at the post-intervention (week 8) and follow-up (week 20) assessment stages, participants assigned to access the intervention immediately will have fewer anxiety and depressive symptoms than those assigned to the wait-list control group.

In addition, it is hypothesised that at post-intervention and follow-up participants allocated to the intervention group will have less psychological distress, reduced internalised homophobia, greater life satisfaction, wellbeing, greater perception of control over their health, and more positive help-seeking attitudes than participants in the wait-list control group.

### Statistical analyses

All scales used in the analysis will be validated using confirmatory factor analysis and reliability analyses. The intervention and wait-list control group will then be compared in terms of baseline measures for symptoms, health behaviour and wellbeing as well as possible confounds such as accessibility/remoteness and gender. Two multi-level modelling analyses will then be conducted. In the first analysis, the intervention and control group will be compared in relation to pre, post and follow-up changes for symptoms, health behaviour and wellbeing. In this analysis, significance levels will be adjusted to reflect the multiple primary outcome measures. In the second multi-level analysis only the intervention group will be considered, with fortnightly changes in the K10 compared in terms of the specific symptom-based programs that were completed by each participant during the previous fortnight (or previously in their intervention).

### Sample size and power calculations

GPower [60] analysis indicates that to detect a small to medium effect size (*f* (V) test = .15) with power of at least .80 and an alpha level of .05, a sample size of 38 participants per condition is sufficient, assuming that all these participants complete all questionnaires. Because drop-out rates at follow-up stage have been reported as high as 70% [61] in trials of Internet-based mental health interventions, it is proposed that a total of 200 young people be recruited initially.

### Ethical approval and trial registration

The proposed study will be conducted in accordance with the ethical guidelines outlined in the National Statement on Ethical Conduct in Human Research [62]. Ethical approval for this study was granted by the Human Research Ethics Committees of Swinburne University of Technology (SUHREC Project 2011/109) and Federation University Australia (B11-077) in June 2011. The trial has been registered with the Australian New Zealand Clinical Trial Registry since 7 July 2011: ACTRN12611000700932.

## Discussion

This paper describes the study protocol for the development and evaluation of a mental health prevention program for SSAYA. Despite having a higher incidence of mental health problems than adults 25 years of age or over [39], only 1 in 4 young people with mental health problems receive professional help and there is a shortage of trained health professionals available to help them [63]. If successful, this online intervention will represent a significant step forward in terms of prevention and early intervention of mental health problems in SSAYA, and addressing barriers associated with stigma and geographic, social and financial isolation. The anticipated strengths and potential limitations of this study are detailed below.

The proposed program is unique and sophisticated, not only in targeting the mental health needs of SSAYA but in tailoring the program content to gender and symptom profile. The personalisation of this program and online nature may help to make it more attractive and engaging for young people than traditional face-to-face alternatives. Moreover, the integrated nature of the intervention enables the simultaneous treatment of multiple mental health symptoms within one program. This is a unique feature of the program, streamlining health care for the young person, as well as acknowledging the uniqueness of each user. This is expected to be a particularly important outcome for SSAYA in rural or regional areas. We expect the findings will enhance our understanding of the impact of internalised homophobia on recovery-related outcomes. We also anticipate that the use of the control condition will enable greater insight into the unique contribution of this program compared to standard mental health and wellbeing services available to SSAYA.

Despite these strengths, three potential limitations of this study design should be acknowledged. Firstly, each participant with a different set of symptoms identified at pre-intervention assessment will get different intervention content due to their receiving content for a different number and combination of types of symptoms when they do the *Out & Online* program. The main comparison with the wait-list control group cannot take these different interventions into account but follow-up statistical analyses will target the efficacy of interventions for each symptom type in isolation and in combination with other symptom types using regression analyses.

Secondly, participant drop-out is a potential challenge, particularly given the self-help nature of the intervention [64]. To enhance motivation, the program modules are deliberately brief and incorporate interactive exercises, videos, audios, pictures and downloadable tip sheets. Contact details are provided on the website in case of questions or technical details. To encourage survey completion automated Emails are sent to participants and they are required to complete the K10 every 2 weeks while in the program in order to regain access to content. Sample size and power calculations have taken into account potential attrition and a conservative intention-to-treat analysis will be complimented with a longitudinal multi-level analysis to ameliorate the effects of drop-out at follow-up.

A third potential limitation is that the intervention is being compared to a wait-list control group, whereby participants will be able to access alternate evidence-based mental health services, including CBT. Information will be collected from participants about mental health service usage and the potential impact of this on outcomes will be statically controlled for.

In summary, SSAYA are at greater risk of poor mental health outcomes compared to their heterosexual peers. The provision of a preventive online mental health and wellbeing program, tailored to the gender and symptoms of the participant, may provide a cost-effective, anonymous and flexible medium for improving mental health outcomes for these individuals.

### Trial status

Recruitment began on 23 July 2014 and will continue until February 2015.

## Author information

JA is a health psychologist and research fellow at the National eTherapy Centre, Swinburne University of Technology. BK is a clinical psychologist, Professor of Psychology and eHealth at Federation University Australia, a Visiting Fellow at the ANU and an Adjunct Professor at Swinburne University of Technology. SM is a psychologist, Professor of Psychology and Head of Psychology at Federation University Australia. DA is a clinical psychologist and Associate Professor of Psychology at Deakin University. MM is a clinical psychologist and lecturer in psychology at Federation University Australia. DM is a statistician and Associate Professor in Statistics, Data Science and Epidemiology at Swinburne University of Technology. BM is a provisional psychologist, research assistant and PhD (clinical psychology) candidate at Swinburne University of Technology.

## Abbreviations

CBT: Cognitive behaviour therapy
PTSD: Posttraumatic stress disorder
RCT: Randomised controlled trial
SAD: Social anxiety disorder
SSAYA: Same-sex attracted young adults.
